# Organoids and chimeras: the hopeful fusion transforming traumatic brain injury research

**DOI:** 10.1186/s40478-024-01845-5

**Published:** 2024-08-30

**Authors:** Cristina Bellotti, Samudyata Samudyata, Sebastian Thams, Carl M. Sellgren, Elham Rostami

**Affiliations:** 1 Department of Neuroscience, Karolinska Institutet, Stockholm, Sweden; 2 Department of Physiology and Pharmacology, Karolinska Institutet, Stockholm, Sweden; 3 Centre for Psychiatry Research, Department of Clinical Neuroscience, Stockholm Health Care Services, Karolinska Institutet, and Stockholm Health Care Services, Stockholm, Sweden; 4 Department of Medical Sciences, Section of Neurosurgery, Uppsala University, Uppsala, Sweden

## Abstract

Research in the field of traumatic brain injury has until now heavily relied on the use of animal models to identify potential therapeutic approaches. However, a long series of failed clinical trials has brought many scientists to question the translational reliability of pre-clinical results obtained in animals. The search for an alternative to conventional models that better replicate human pathology in traumatic brain injury is thus of the utmost importance for the field. Recently, orthotopic xenotransplantation of human brain organoids into living animal models has been achieved. This review summarizes the existing literature on this new method, focusing on its potential applications in preclinical research, both in the context of cell replacement therapy and disease modelling. Given the obvious advantages of this approach to study human pathologies in an in vivo context, we here critically review its current limitations while considering its possible applications in traumatic brain injury research.

## Introduction

Traumatic Brain Injury (TBI) poses a significant global health challenge, affecting millions annually and ranging in severity from mild to severe, as assessed by the Glasgow coma scale [34, 114, 115]. While mild cases often present a full recovery, moderate to severe TBI can result in devastating consequences with persistent and pronounced impairments in cognitive and emotional functioning. On the other hand, it is important to note that even mild TBI significantly raises the risk of sustained neurocognitive and neurobehavioral symptoms [91]. TBI is a heterogeneous disease with heterogeneous outcomes and despite extensive research, a substantial portion of inter-individual variability in TBI outcomes remains unexplained [63]. It is noteworthy that individuals with seemingly similar injuries can exhibit diverse outcomes, highlighting the intricate interplay of factors influencing recovery. Notably, genetic factors contribute significantly to this variability, with approximately 26% of the differences in TBI outcomes attributed to genetic influences [49]. This underscores the necessity for a comprehensive understanding of how genes influence outcomes in TBI, as such an understanding is crucial for therapeutic development and intervention strategies.

Despite advancements in acute care and neurointensive treatments, identifying effective long-term interventions has remained a daunting task, evident from more than 40 failed clinical trials in the field [107]. The failed pharmacological clinical trials have been based on results from animal models, underscoring the difficulty in translating these results to human studies [94, 100, 106]. This emphasizes the crucial requirement for alternatives to traditional animal models to accurately mimic human TBI for both research and pharmacological testing purposes. Brain organoids are self-organized multicellular constructs that can be generated from human induced pluripotent stem cells (hiPSCs) [58]. These complex cellular systems can be grown in culture over long time-periods and have been shown to remarkably capture both structural and functional features of the living human brain. Nonetheless, available in vitro brain organoid models still lack micro-vascularisation, and foremost reflect the early time-points in brain development [36]. To address these limitations, chimeric models have recently been developed that relies on transplanting human brain organoids into living brain of rodents [68, 86] or non-human primates [55]. Such models could offer an advanced platform for studying brain injuries, particularly using human- and eventually patient-derived brain organoids. Moreover, it provides an opportunity to assess cell replacement therapy and conduct preclinical tests for neuroprotective drugs. Additionally, it contributes to the identification of genes and networks responsible for individual variations in recovery from brain injury, thereby paving the way for the development of novel therapeutic approaches.

This review first gives a brief introduction to TBI pathology and the development of organoid technology. It then focuses on relevant studies using human brain organoid transplantation, critiques their limitations, and advocates for standardized reporting to accelerate progress in the field and facilitate the use of shared data.

## TBI pathology

**Fig. 1:**
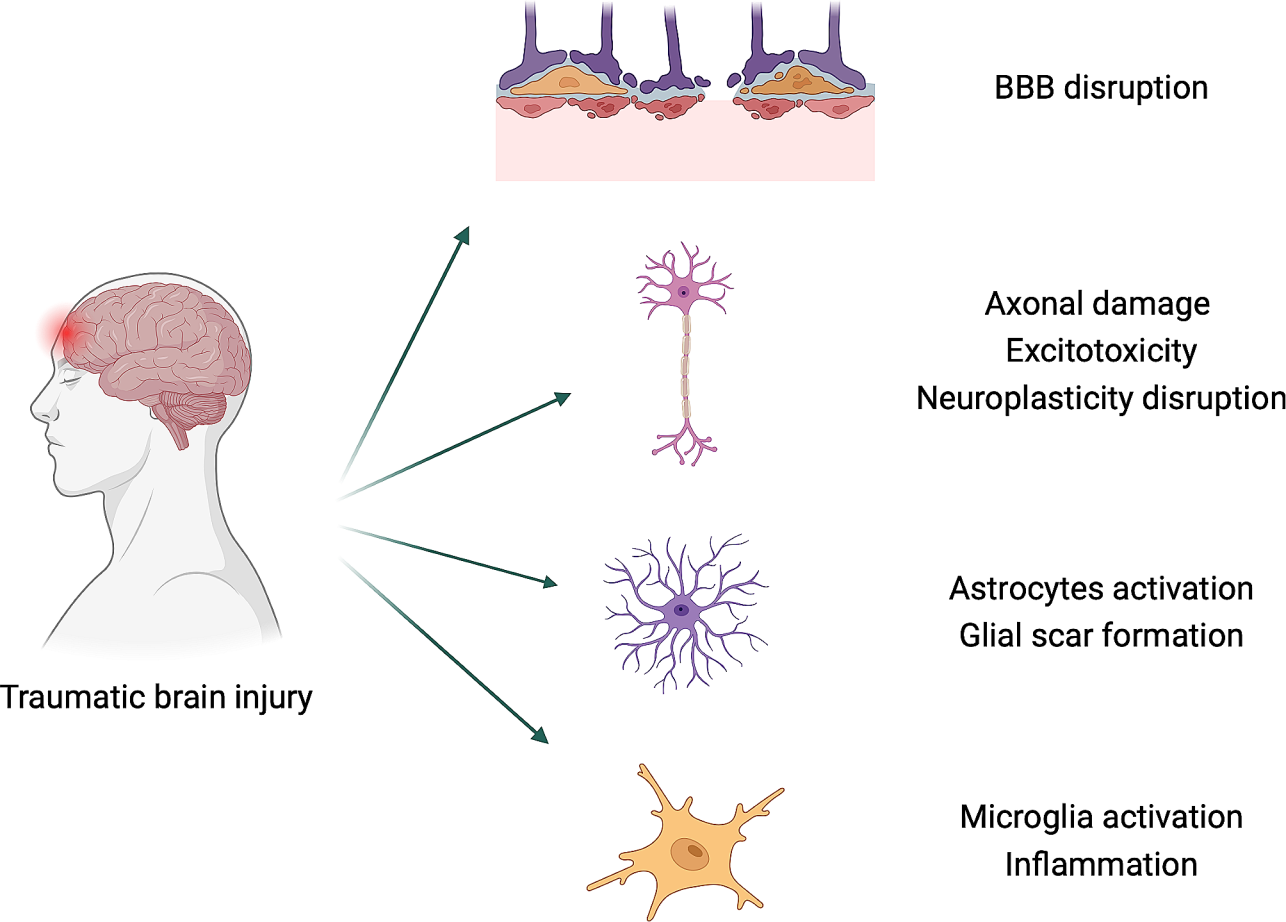
TBI pathology. Illustration of some of the processes that take place during the secondary injury. All cell types in the brain are involved in TBI pathology. BBB: blood brain barrier. Image created with BioRender

TBI is a dynamic disease with the initial primary injury triggering secondary processes that can progress over time [65, 75]. The primary injury involves the immediate physical damage caused by the impact. This damage can manifest as focal injuries, localized at the site of impact, or diffuse injuries, affecting multiple areas of the brain due to rapid acceleration and deceleration forces [3]. Subsequently, secondary injury mechanisms come into play, occurring in the minutes to weeks following the primary injury (Fig. 1). Excitotoxicity, a process triggered by excessive release of neurotransmitters like glutamate, leads to neuronal overstimulation, synaptic loss and eventual cell death [6]. Oxidative stress further complicates matters, as the brain generates reactive oxygen species and free radicals, damaging cell membranes, proteins, and DNA [18].

**Fig. 2:**
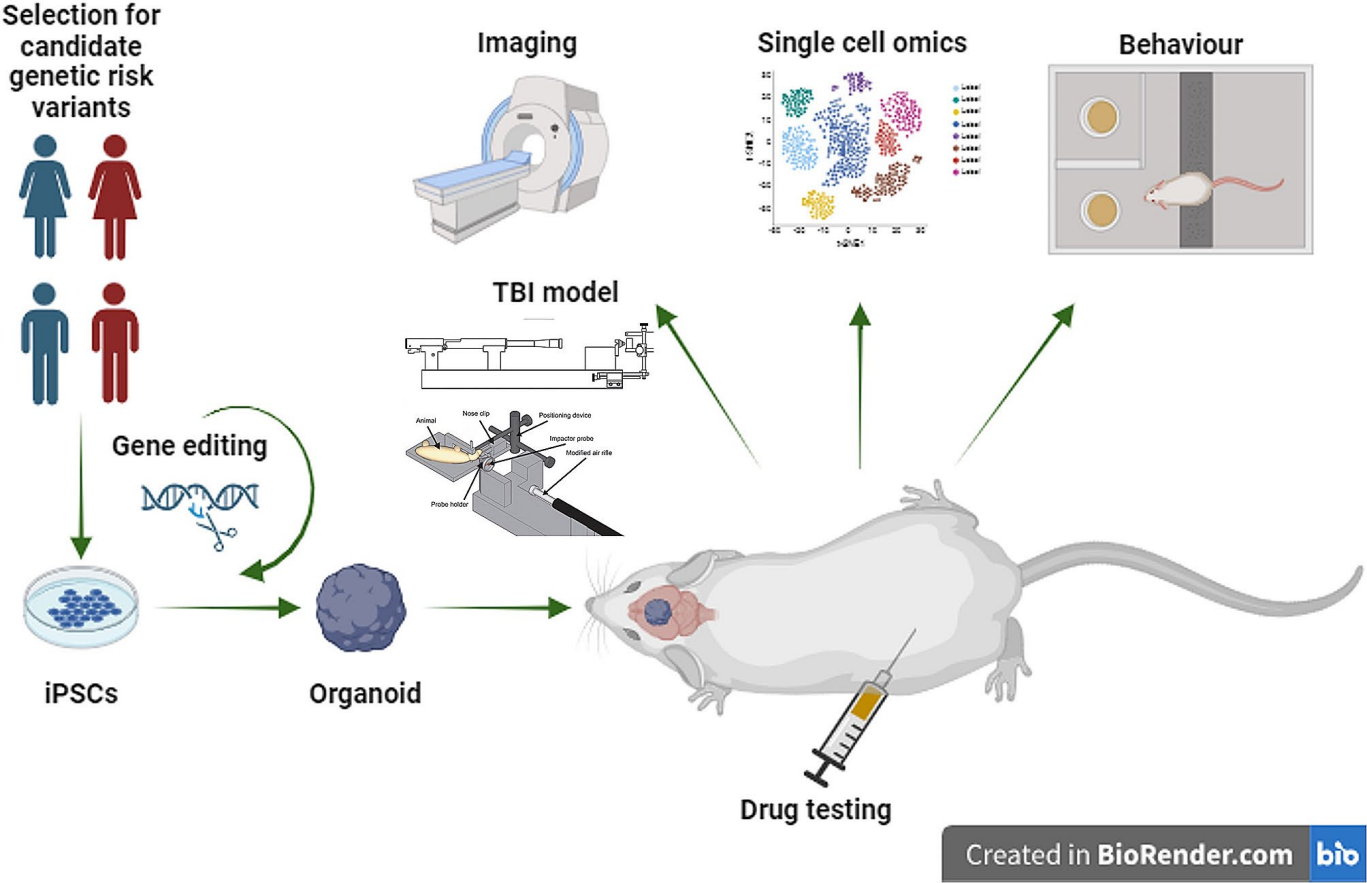
Potential experimental set-up for the study of TBI in in vivo human brain organoids. Genetic influence on TBI outcome could be studied by using iPSCs from individuals carrying candidate risk variants. Targeted gene editing could be used to modify candidate genes. iPSCs-derived organoids would be transplanted in a rodent host and allowed to differentiate and integrate. The organoid would be subjected to TBI using an experimental TBI model and a variety of experimental readouts would be used to estimate the response of the organoid to TBI. At the same time, the model could be exploited to test the efficacy of potential therapy agents in preventing secondary injury in the human tissue. Image created with BioRender

Inflammation, a natural immune response, is activated post-TBI [72]. While inflammation is essential for clearing damaged cells, excessive or prolonged inflammation can cause additional tissue damage. Apoptosis and necrosis are common outcomes, contributing to the formation of lesion areas and loss of functional neuronal circuits.

The blood-brain barrier, which normally restricts the entry of harmful substances into the brain, can be compromised after TBI [33, 103]. This disruption exacerbates inflammation and neuronal damage. Furthermore, disruptions in neuroplasticity [19, 90, 95, 119], i.e., the ability of the brain to reorganize and form new neuronal connections, can impair the capacity of the brain to adapt and recover after injury. Finally, TBI can initiate or accelerate chronic neurodegenerative processes, leading to long-term cognitive and motor impairments [70, 108].

Hence, understanding this complex pathophysiology is crucial for developing targeted therapies that can prevent or reverse these processes, and ultimately improve outcomes for individuals impacted by TBI.

Several experimental TBI models have been developed in rodents [21, 22, 87, 126], which have played a pivotal role in understanding the pathophysiology of TBI and have offered promising therapeutic avenues. Despite these insights, translating pre-clinical findings into successful clinical trials has proven challenging. Stratification of TBI patients based on severity, rather than the underlying pathology, has likely contributed to this [67]. On the other hand, questions have been raised about intrinsic differences between rodent and human responses to injury and therapeutic interventions. Therefore, utilizing more humanized models to study injury responses and therapeutic possibilities has become increasingly appealing. These models could not only offer insights into patient-specific injury responses but also serve as platforms for personalized and precision medicine, revolutionizing the approach to TBI research and therapy.

## Organoid development: towards personalized medicine

Organoids are self-organized 3D tissue culture systems, mostly derived from embryonic or induced pluripotent stem cells, that mimic organ structure and function in vitro. Since the development of intestinal epithelium organoids in 2009 [92], protocols have been established for the growth of organoids modelling a variety of tissues, such as the optic cup [28], brain [59], liver [113], kidney [112] and lung [27]. Specifically, brain organoids recapitulate the emergence of ventricular zone (VZ)-like structures consisting of radially organised neuroepithelial progenitors which over time differentiate to give rise to neurons and glia. There are two major approaches to brain organoid modelling, and they are defined by reliance on either intrinsic cues generated by stem cell aggregates called embryoid bodies to achieve neuroectodermal fate, or addition of external factors like morphogens and small molecules to induce or restrict specific fates [71, 130]. Accordingly, the former approach results in more complex and variable unguided- or whole brain- or cerebral organoids (COs) with a mix of regional identities, while the latter produces organoids with more homogenous cell populations that resemble specific brain regions such as forebrain [4, 88], midbrain [47], hindbrain [29] etc.

Due to organoids achieving a higher tissue complexity than 2D cell cultures, they allow researchers to better model tissue development and diseases [101]. Indeed, COs have been used to study a variety of brain disorders in vitro [2, 109, 131], including microcephaly [59], autism [61, 69], Alzheimer [62, 83], Zika virus [56, 82] and Sars-CoV-2 infection [43, 79, 84, 89]. In addition, organoids could also be used for drug screening [53], especially for pathologies for which animal models fail to reliably replicate human phenotypes. The fact that pluripotent stem cells can be obtained by reprogramming of somatic cells from human subjects [78, 111] makes it possible to preserve genetic background of the subject while also obtaining patient-specific organoids, which holds great potential for personalized medicine. A particularly interesting application of this would be the possible use of organoids in regenerative medicine and for autologous transplantation. The feasibility of transplantation of organoids in animal hosts has been demonstrated for several tissues [76, 113, 116, 117, 122] including structures of the central nervous system (CNS). For example, it has been employed for repair of spinal cord lesions [57, 127] and as replacement therapy for retinal degeneration [73, 99, 123], with transplantation of retinal sheets reaching clinical trial stage [66].

In recent years, some groups have used COs to model aspects of primary TBI. Silvosa et al. exposed COs to pressure waves of different frequencies and amplitudes in a tabletop blast device [102]. Results highlighted how waves at pressure injury threshold can transiently disrupt electrophysiological activity without causing cell death. In another study, Beltrán et al [10]. subjected COs to different rates of mechanical strain by uniaxially compressing them, and RNA-seq was used to identify pathways differentially expressed in different injury conditions. In an interesting set-up, Ramirez et al [85]. devised a way to use controlled cortical impact (CCI) equipment on organoids. Using an agarose-gelatine mixture, they developed a “phantom brain” which replicates mechanical characteristic of the mouse brain and enclosed it in a real mouse skull. The CO was then deposited on the phantom brain and subjected to CCI. Immunohistochemistry revealed that CCI can induce astrogliosis, neuronal damage and cellular apoptosis in COs.

While these in vitro studies are clearly informative, COs cannot completely replicate the complexity of the CNS environment, including the biomechanics of the skull, the hemodynamic changes of intracranial pressure and the cerebral blood flow mediating effects of systemic responses to injury. Furthermore, it is not possible to model the immunological response to injury, which is a fundamental player in primary and secondary TBI. In addition, COs tend to develop a necrotic core when they are grown to larger sizes and over longer time-periods, probably due to lack of vasculature and insufficient diffusion of nutrients and oxygen. To overcome this issue, Mansour et al. [68]. transplanted human COs into the cortex of adult immune-supressed mice and observed their survival and integration into the host brain. In the following years, several studies replicated and expanded their findings, creating what is now a solid body of scientific literature on this topic (Table 1). Results are summarized in the following sections, with a focus on the potential of this protocol for cell replacement therapy and disease modelling, particularly for TBI.

**Table 1:** Relevant parameters of published CO transplantation studies

Author	Year	Animal	Animal age	Sex	Lesion type	Time of transplant	Brain area of transplant	Follow-up
Pham et al.	2018	NSG mice	2 months	Male	Direct removal of brain tissue ∼ 2 × 2 × 2 mm	Immediately after injury	Cortex	2 weeks
Mansour et al.	2018	NOD-SCID mice	6–10 weeks	Female (validated in male)	Aspirative cavity	Immediately after injury	Retrosplenial cortex	8 months
Daviaud et al.	2018	CD1 mice	P8-P10	Male and female	Removal of brain tissue (∼ 1 mm^3^) with stab knife	Immediately after injury	Frontoparietal cortex	4 weeks
Wang et al.	2019	Sprague-Dawley rats	----	Male	MCAO + biopsy punch	6 h/24 h/7 days after injury	Motor cortex	28 days
Kitahara et al.	2020	SCID mice; Macaca fascicularis	Mice: 7 days/6 weeks Monkeys: 3 years	Mice: male and female (7d), male (6w) Monkeys: male	Aspirative cavity	Immediately/7 days after injury	Mice: frontoparietal/frontal cortex Monkeys: precentral cortex	12 weeks
Wang et al.	2020	Sprague-Dawley rats	----	Male	Biopsy punch	Immediately after injury	Motor cortex	56 days
Shi et al.	2020	NOD-SCID mice	8 weeks	----	Aspirative cavity	Immediately after injury	S1 cortex	60 days
Dong et al.	2020	SCID mice	6–8 weeks	----	No lesion		Medial prefrontal cortex	5 to 6 months
Bao et al.	2021	SCID mice	8 weeks	----	CCI	7 days after injury	Parietal cortex	70 days
Revah et al.	2022	RNU nude rats	P3-P7	Male and female	No lesion		S1 cortex	8 months^a^
Wilson et al.	2022	NOD-SCID mice	8–12 weeks	Female	Aspirative cavity (∼ 1 mm diameter)	Immediately after injury	Retrosplenial cortex	11 weeks
Huang et al.	2022	SCID mice	4–5 weeks	Female	No lesion		Corpus striatum	60 days
Kim et al.	2022	C57BL/6 J mice	----	Male	CCI + biopsy punch	7 days after injury	Cortex above hippocampus	14 days
Jgamadze et al.	2023	Long Evans rats	8–12 weeks	Male	Aspirative cavity	Immediatly after injury	Visual cortex	3 months
Zheng et al.	2023	SCID mice	8–12 weeks	----	6-OHDA injection	4 weeks after injury	Corpus striatum	16 weeks
Cao et al.	2023	NOD-SCID mice	7–8 weeks	Male	Photothrombotic stroke	7 days after injury	Forelimb motor/parietal cortex	180 days
Schafer et al.	2023	NOD-SCID mice	6–10 weeks	Female (majority)	Aspirative cavity	Immediately after injury	Retrosplenial cortex	24 weeks
Cao et al.	2023	NOD-SCID mice	7–8 weeks	Male	Photothrombotic stroke	7 days after injury	Forelimb motor cortex	180 days
Chen et al.	2024	RNU nude rats	P3-P7	Male and female	No lesion		S1 cortex	9 months

“No lesion” indicates that the organoids were transplanted by needle injection, without further damage to host brain. In the “Follow up” column we listed the latest time points at which experiments were performed for each study. NSG: NOD-SCID gamma. NOD-SCID: nonobese diabetic - severe combined immunodeficiency. MCAO: middle cerebral artery occlusion. 6-OHDA: 6-hydroxydopamine. ^a^: survival of grafted animals was evaluated up to 12 mpt

## Organoid integration in the host brain

### Vascularization

As mentioned above, lack of vasculature in COs is an obstacle to their long-term growth. Consequently, several attempts have been made to establish protocols for the generation of vascularized COs [128]. The first ever reported in vivo CO transplantation was performed by Pham et al [81]. using a CO coated with iPSC-derived endothelial cells from the same patient. Two weeks after transplantation, the organoid had survived and was penetrated by vascular structures of human origin, although connection with the host vasculature was not tested. A similar approach was used by Shi et al. [98] who obtained vascularized COs by coculturing human stem cells with human umbilical vein endothelial cells. After transplantation in the mouse cortex, steady blood flow could be detected in the COs, which contained both human and mouse endothelial cells. Compared to control COs, the vascularized ones appeared to have lower cell death 60 days post transplantation (dpt). In contrast, other transplantation experiments using COs without coating of human endothelial cells have demonstrated host CD31 + microvasculature invading the organoid graft as early as 7–10 dpt, leading to extensive vascularisation by 14 dpt [20, 68], with observed blood flow in the grafts [68, 125], and resulting in robust integration and graft survival ≥ 80% and up to 8 months [46, 55, 68, 86].

### Differentiation

In most of the studies presented above, COs were transplanted after being cultured for 40–60 days in vitro (DIV). Afterwards, COs continued to progressively differentiate in vivo: the percentage of human cells expressing histological markers characteristic of progenitor cells, like Sox2, Pax6 and Nestin, also decreased over time [46, 68, 120, 121]. On the other hand, markers indicating neuronal commitment/differentiation, such as Tuj1 and NeuN, were expressed by a progressively higher percentage of grafted cells [46, 68, 121]. An exception is the study by Daviaud et al [20]. that did not observe a decrease in Sox2 expression, although the observation period, from 2 to 4 weeks post transplantation (wpt), might have been too short. Further, transplanted COs also displayed increased expression of markers for deep- and surface-layer neurons over time (CTIP2 and SATB2) [46]. Transcriptomics analyses then confirmed a progressive maturation of transplanted COs, to a large degree mimicking foetal human brain development [93]. Given that COs cultured in vitro foremost have been shown to recapitulate early brain development, and the high costs of keeping COs in in vitro culture, xenotransplantation of COs holds promises for more accurate modelling of later stages of brain development.

A minor percentage of the cells in COs differentiate into glial cells in vivo. While GFAP and Olig2 were often used as markers for astrocytes and oligodendrocytes, they are not exclusively expressed in these cell types and cellular identity was rarely confirmed with other markers [68, 98]. GFAP expression in the graft was reported to increase over time after transplant [20]. PDGFRα, a marker of oligodendrocyte precursor cells, could also be detected in grafted organoids [26, 86]. On the other hand, staining for myelin basic protein revealed scarce or no expression [26, 68, 98], suggesting absent or late-onset myelination in the COs. There were contrasting findings on the origin of GFAP + and Olig2 + cells in the transplant area: while some observed mainly human cells [14, 20], others reported a majority of infiltrating host cells [46, 98]. Single cells RNA-seq of organoids 2 [42] and 8 [86] months post transplantation (mpt) confirmed the presence of clusters pertaining to astrocytes and oligodendrocytes lineage cells.

While neurons, astrocytes and oligodendrocytes all derive from neuroectoderm, microglia originate from erythromyeloid progenitors (EMP) in the yolk sac [35, 51]. Most guided organoid protocols inhibit non-neuroectodermal fate via dual SMAD signalling, thus obtaining microglia-free organoids, even if microglia-like cells were reported to spontaneously develop in undirected approaches with lowered dose of neuroectoderm stimulant, heparin and delayed Matrigel embedding [77]. Several transplantation studies found that Iba1 + cells in the grafted organoids were exclusively of host origin, independently from the differentiation protocol used before transplant [42, 68, 86]. Host Iba1 + cells were detected in the graft as early as 2 wpt [20]. To obtain immune-competent COs in vivo, Schafer et al [93]. derived forebrain organoids and EMPs from the same stem cell line and co-cultured them together before transplantation into mice. The EMP in the graft differentiated into Iba1 + cells with a ramified morphology and expression of homeostatic microglia markers. Human microglia density in the transplanted organoid also increased over time.

### Graft-host synapses, axonal projections and functional integration

Colocalization of puncta expressing pre- and post-synaptic markers, such as Synapsin I, Synaptophysin, and PSD95, suggested the formation of synapses in transplanted COs [68, 98]. Moreover, staining for human and non-species-specific markers showed the presence of synaptic structures between the graft and the host neurons [68, 125, 132]. Several studies have also reported that axons from organoid neurons extended into the host rodent brain, reaching distal areas, in some cases even traveling in the corpus callosum to reach the hemisphere contralateral to the graft site [14, 68, 132]. Lack of MBP expression around the axons though suggested that the projections were not myelinated [46]. Already at 1 mpt, such long projections could be observed [26], and appeared to increase in density without appreciable change in efferent targets over time [46]. The targets were mostly appropriate for the area in which the CO was transplanted, even if some off-target projections could also be observed [46]. Axons also extended from COs in the brain of non-human primates, even if projections did not reach distal areas as in rodents over the same time period, probably due to the longer distances they need to cover [55]. Presence of active synaptic connections with the host brains and identity of afferent/efferent brain areas were also confirmed with anterograde and retrograde synaptic tracing [26, 86, 132]. For example, polysynaptic tracing with herpes simplex virus allowed scientist to demonstrate indirect connections between the rat retina and the organoid grafted in the visual cortex [46].

Electrophysiology has extensively been used to test neuronal functions in transplanted COs. Spontaneous and evoked action potentials have been measured in patch-clamp experiments on transplanted CO neurons in ex-vivo brain slices [5, 14, 132]. Moreover, stimulation of fibres from host brain putative afferent areas produced post-synaptic responses in human CO neurons [86]. Further, in vivo extracellular recording and two-photon calcium imaging showed the presence of spontaneous synchronized neural activity in the grafts [46, 68, 86]. Changes in some electrophysiological parameters over time also suggested that CO neurons undergo functional maturation in the host cortex and that the process is still ongoing between 3 and 5 mpt [26, 68].

The use of optogenetics has also enabled a more precise characterization of CO integration. By selectively expressing channelrhodopsin 2 in human cells and performing recordings from host cells and vice versa, scientists have been able to confirm the presence of bidirectional synaptic connections between the COs and the host brain [14, 26, 86]. Moreover, specific optogenetic activation of the graft or its efferent fibres has been shown to be associated with changes in local field potentials in different host brain regions [14, 68]. Finally, Revah et al. have used optogenetic stimulation of transplanted COs during training on a modified operant conditioning paradigm and demonstrated that graft activity is able to modulate host behaviour [86].

Functional integration of transplanted COs is also supported by the fact that responses to physiological stimuli can be recorded in grafted neurons. Cells of COs transplanted in brain region S1 have been reported to show increased activity associated with whisker deflection [86], and visually evoked activity could be recorded in the majority of COs implanted into the visual cortex [46]. A subset of neurons also exhibited preferential firing for visual stimuli in a particular orientation, replicating the orientation selectivity typical of visual cortex neurons. Similarly, local field potentials could be detected in response to visual stimuli in COs grafted into the retrosplenial cortex [125]. These responses could be recorded already 3 wpt, but were stronger at > 50 dpt, again suggesting circuit maturation. It is important to note that in all these studies, organoid electrophysiological responses resembled those of the host neurons, although some differences could still be detected. For example, CO spontaneous activity was more inhibited than that of surrounding cortex by isoflurane anaesthesia 3 wpt [125].

### Comparison to in vitro organoids and single cell transplant

Until now, research in cell replacement therapy for neurological disorders has mostly focused on the use of single cell suspensions [30]. However, transplanted Sox2-positive neural progenitor cells (NPCs) shrank considerably from 2 to 4 wpt while COs remained stable (in identical cortical location) [20]. Mirroring this, dissociated NPCs showed abundant vasculature at 2 wpt, but at 4 wpt the number of CD31 + blood vessels were lower when compared to transplanted COs [20]. Additionally, while there was no significant change in the percentage of apoptotic cells between the two types of graft, there was an increase in host Iba1 + cells with hypertrophied morphology, a decrease in DCX + neuroblasts, and correspondingly no presence of NF-H + mature neurons, in NPC transplants at 4 wpt, thus indicating beneficial effects of the human multicellular 3D microenvironment as offered by COs. This is also supported by the fact that when COs are dissociated and transplanted as single cells, they achieve less cell survival and graft volume than their intact counterparts [14, 46].

In several cases, transplanted COs have also been directly compared to age-matched COs kept *in vitro.* Cell death was greatly reduced in grafted COs, particularly at later time points, with a higher percentage of cells expressing mature neuronal markers in transplanted COs [68]. Single nucleus RNA-seq also supported a more mature transcriptional profile with especially oligodendrocytes-related clusters being detected exclusively in transplanted COs [86]. Correspondingly, neurons were morphologically and electrophysiologically more developed in the transplanted COs. For transplanted immune-competent organoids, recent findings suggests that microglia ramification and expression of homeostatic markers is enhanced in vivo, while cellular activation and stress-related responses decrease [93].

In sum, these results appear to indicate that transplantation of COs can be advantageous for applications within the field of regenerative medicine compared to single cell transplantation and can serve as a superior model of the human brain compared to in vitro COs.

### Organoid transplantation in preclinical research

In addition to monitoring the integration and differentiation of COs after transplantation into the host brain, some scientists have starting to explore potential applications of the model. We report their findings in the following sections, highlighting how certain results could be translated to TBI research.

### Organoids as a cell replacement therapy

Most of the studies listed above have employed tools to create a cavity in the host brain to accommodate transplantation [44]. Consequently, researchers have investigated if COs are able to not only integrate in the host tissue but also repair the lesion. In many cases, the injury has simply been removal of cortical tissue, which, while traumatic in itself, lacks the features of real-life TBI offered by traditional experimental models. Nonetheless, some of these studies can still provide us information on the potential of COs in regenerative medicine to repair brain lesions. For example, it would be important to ensure that the graft itself does not worsen the inflammatory response caused by injury. Jgamadze et al. [46] examined the response of the host tissue to the CO graft and compared it to animals which received an injury without transplantation. They observed decline in GFAP + cells in the tissue surrounding the graft over time which suggested a reduction in astrogliosis. Notably, these cells did not form a glial scar and were present in fewer numbers as compared to the injury-only controls. This further suggests that the graft may contribute to minimizing astrogliosis, highlighting a potential therapeutic effect in modulating glial response to injury. While Iba1 + microglia exhibited a decrease over time, there was no significant difference compared to controls. However, activated microglia (defined as CD68+) demonstrated higher density in the graft-receiving animals as compared to those with injury only. This observation suggests the presence of low but persistent inflammation in the graft recipients, indicating a nuanced immune response that warrants further investigation.

Several studies, summarized in the following subsections, utilized injury models that closely mimic relevant pathologies to assess the therapeutic potential of organoids in these contexts.

#### TBI

In Wang et al. [121] a biopsy punch in the rat motor cortex was used as a model of TBI. The expression of inflammatory cytokines was then similar when COs were transplanted as compared to sham and injury-only conditions. CO transplantation also resulted in increased neurogenesis in the cortex peripheral to the transplant and in the ipsilateral subgranular (SGZ) and subventricular zones (SVZ) of the host. In addition, some synaptic proteins and growth factors were upregulated in the ipsilateral hippocampus in grafted animals. In particular, brain-derived neurotrophic factor (BDNF) and nerve growth factor (NGF) were downregulated following injury compared to shams but were upregulated only in transplanted animals by 28- and 56-days post-injury (dpi), respectively. This is especially interesting in a TBI context, since BDNF has been the focus of extensive research in this field [40]. Further, behavioural tests showed a positive effect of CO transplantation on motor performance already from 5 dpi, while the modified neurological severity scores were back to sham levels by 21 dpi. Impairment in the beam walking test, while being consistently reduced compared to the injury-only group, persisted until the end of experiments (42 dpi).

Only two studies, conducted by Bao et al. [5] and Kim et al. [52], have utilized a traditional experimental TBI model (CCI). Notably, in the latter study, a biopsy punch was used after the initial CCI to create a cavity for the organoid, although it is not clear if the control group (TBI without transplant) also received this treatment or not. Consequently, it is difficult to distinguish between the effect of the CCI and the successive tissue lesion. In addition, the follow-up in this study was only 2 wpt. Animal survival up to 70 dpi was not influenced by CO transplantation [5]. As previously reported, there was some evidence of increased neurogenesis in the SVZ and dentate gyrus of injured animals receiving COs as compared to the injury-only group [52]. In addition, fewer apoptotic neurons were observed in the hippocampus and peri-lesional cortex of grafted animals. In accordance with Jgamadze et al., the numbers of Iba1^+^ microglia at the lesion periphery were not influenced by the CO presence. Finally, grafted animals showed improvements in spatial learning and memory compared to injury-only controls, measured with the Morris water maze and passive avoidance test [5].

In summary, these publications suggest that CO transplantation could have some positive effect on recovery following TBI, but further studies are needed.

#### Other pathologies

Two previous studies have reported use of COs to repair stroke lesions in rodents. Wang et al. [120] performed CO transplantation in a middle cerebral artery occlusion rat model. A biopsy punch was used to create a cavity for the organoid in the motor cortex (injured controls received both the occlusion and the biopsy punch). Instead, Cao et al [13, 14]. transplanted COs directly into the cortical ischemic lesion developed in a photochemical stroke mouse model [105]. As seen in TBI, CO transplantation was associated with increased neurogenesis in SVZ and SGZ of the host, both ipsilateral and contralateral, with most of the new cells being of endogenous origin [120]. The majority of the newly formed cells in the peripheral area of the transplant were instead of human origin. Increased expression of synaptic markers in the transplant periphery and ipsilateral SGZ of grafted vs. injury-only animals was also detected 7 dpt, but it was not tested at later time points. The area of the ipsilateral cortex expressing MBP and NF-H was increased in animals receiving transplantation, as was the number of new cells expressing markers of oligodendrocyte lineage. In addition, newly born endothelial cells were more numerous in the transplantation periphery of grafted animals. However, the identity of the cells expressing these markers was not verified. Part of these results could likely be explained by increased tissue survival in the grafted animals, as the infarct volume and the number of apoptotic cells in the transplantation periphery were reduced in this group. As observed in previously mentioned studies, neuroinflammation at the transplant site seemed unaffected by the presence of the CO, although there were indications that its cells might contribute to glial scar formation at the infarct border. Finally, neurological motor function was improved in grafted vs. injury-only animals as early as 2 dpt. Cao et al. also observed greater performance recovery of transplanted animals in several sensorimotor tests, reaching levels similar to sham at the end of experiments (150 dpt). Notably, selective chemical silencing of the CO cells significantly worsened performance of grafted animals in 3 out of 4 behavioural tests, indicating that human neurons directly participated in sensorimotor function [14]. While the pathological processes in TBI and stroke differ in many aspects, the fact that several results could be replicated in both conditions is a positive signal for the regenerative potential of CO transplantation.

Finally, COs have also been used to restore function in Parkinson´s disease (PD) [132]. 6-hydroxydopamine was used to cause loss of midbrain dopaminergic neurons in mice, and COs were transplanted into the striatum after 4 weeks. In this study, mRNA expression of two inflammatory cytokines (*IL-1β* and *IL-6*) was reduced in the striatum of transplanted mice as compared to lesion-only animals, and expression of a cytoprotective gene (*Hmox1*) was increased. It is important to note that this was the only study in which midbrain organoids were used and the lesion and transplantation protocol were considerably different from the experiments reported before; this could be the cause for apparent discrepancies. Over time, grafted animals partially recovered motor function in several behavioural tests, while lesion-control mice did not. These results underline the versatility of COs transplantation as a cell replacement strategy which can be adapted to different brain areas and conditions. Considering the heterogeneity of areas that can be involved in TBI, different COs transplantation protocols could be established and applied on a case-by-case basis.

### In vivo organoids for modelling of human pathologies

As mentioned above, xenotransplantion of COs could give us the rare opportunity to study human tissue inserted in a complex in vivo system. Despite this potential, only a few attempts have been performed so far to model human pathologies.

Revah et al. [86] generated COs from patients affected by Timothy Syndrome, a severe neurodevelopmental disease. Eight mpt in rat, neurons of these COs recapitulated alterations in their dendritic morphology as compared to control COs. Their electrophysiological properties were also altered. Notably, these phenotypes were not evident in COs grown only *in vitro.* In a subsequent study [17], this model was used to test a novel therapeutic strategy for Timothy Syndrome, demonstrating its potential in preclinical research.

Schafer et al. [93] showed that the human microglia in their in vivo transplanted neuroimmune COs reacted to both a focal laser lesion and systemic inflammation. They then derived neuroimmune COs from hiPSC of subjects with autism spectrum disorder and observed an increase in reactive microglia compared to controls 12 wpt. Moreover, the change in morphology was observed also when the microglia was differentiated from hiPSC of healthy subjects and grown within ASD COs, demonstrating that the altered brain microenvironment is sufficient to induce the phenotype. Including human-derived microglia in the in vivo CO model is also highly relevant for several other CNS pathologies that involve this cell type, as human and rodent microglia exhibit notable differences. Transcriptomic and epigenetic analyses have shown species-specific differences in microglia-enriched genes, and that human microglia preferentially express a higher number of genes which are altered in or that are associated to risk alleles for e.g., neurodegenerative diseases [32, 38]. Moreover, very limited overlap has been observed in gene expression changes during aging between mouse and human microglia [31]. One key distinction might lie in the pronounced heterogeneity of human microglia [32]. Considering the significant role of the immune response in many CNS pathologies, understanding these differences is crucial for developing targeted therapies for human neurological disorders. This is true also for TBI, in which the immune system play a critical role in secondary injury [72]. Microglia play a crucial role in recovering from TBI: these immune cells clear debris, aid in remodelling, neurogenesis, angiogenesis, oligodendrogenesis and remyelination, highlighting their vital role in overall CNS repair [64, 124]. This highlights the importance of having the possibility to model in vivo response of specifically human microglia to TBI.

The use of in vivo COs to model TBI, in a set-up in which the injury is performed on the CO first after it has integrated into the host brain, was also suggested in 2020 [45], but yet not applied. An example of experiments that could be performed using this model is reported in Fig. 2. Gene variants that are considered to be candidates for having an effect on outcome variability in TBI [49] could be tested with this platform by developing iPSCs from donors who carry the variants, or by engineering available iPSC lines to include the mutation of interest. Conversely, the candidate variant could be edited out in donor cells to obtain isogenic lines to serve as controls with identical genetic background. These lines would be used to produce COs which then would be transplanted in animal hosts so that they could integrate into their brain. The xenotransplant would allow researchers to use standard TBI models on the organoids, without having to establish new methods to simulate TBI in vitro. In addition, being inserted in a living animal, COs would be affected by the systemic response to injury, which cannot be properly replicated in vitro. It would be possible to test a variety of experimental readouts, including, potentially, behavioural outcomes depending on the brain area in which the organoid would be transplanted. Finally, the model could be used to test potential drug treatments for TBI and their effect on specifically human tissue following different routes of administration, which is not feasible in vitro.

### Considerations: sources of variability in organoid transplantation

Given the novelty of CO transplantation research, protocols are yet to be standardized and the available literature appears to be quite heterogenous. Consequently, we identified several parameters that could influence the experimental outcome.

In most of the listed studies COs were transplanted immediately after producing an injury in the animal brain. Conversely, in both studies testing cell replacement after CCI, transplantation was done 7 dpi. Previous research using embryonic cortical tissue have demonstrated that transplantation can be performed up to 14 dpi [104] and that a 1 week delay between cortical injury and transplantation has a beneficial effect on graft size and integration [80]. Kitahara et al. [55] confirmed that delaying organoid transplantation to 1 week after lesion increased graft survival, size and number of axonal projections. However, this has not been verified in the specific context of organoid transplantation in a TBI model. In stroke, Cao et al. always transplanted organoids 7 days after surgery [13, 14], while Wang et al. tested different time windows: most of their experiments were performed using a 6 h interval between stroke and transplant, but 24 h and 7 days were also probed for their therapeutic potential [120]. A 24 h delay was still associated with some beneficial effects on tissue preservation and behavioural performance, but transplant after 7 days had no benefit for the animals. This discrepancy between studies might be due to the different stroke models employed, which can cause different lesions [105]. This underlines the importance of adapting the transplantation protocol to the injury model in use.

#### Other potentially relevant factors are the age of the animals and of the organoids

So far, most studies have performed the transplantation in adult animals. However, e.g., Daviaud et al [20]. Used mice at P8-P10 and took advantage of the immature immune-system to perform the transplantation without immunosuppression. While comparative studies are still largely missing, it is likely that age of the host animal will influence graft survival and integration. Both Kitahara et al. [55] and Wang et al. [121] have studied how CO DIV influences graft volume or number of surviving cells suggesting that both these outcomes improve when using younger COs at day of transplantation (age and other characteristics of organoids for each study are reported in Table 2)

**Table 2:** Characteristics of organoids used in transplantation studies

Author	Year	Organoid age at transplant	Organoid type	Type of cells	№ of lines	Survival post-implant
Pham et al.	2018	54 days	Undirected, hiPSC-derived EC added at day 34	iPSC	1	
Mansour et al.	2018	40–50 days or 31 days	Undirected	ESC	1	80%
Daviaud et al.	2018	42 days	Undirected	ESC	1	
Wang et al.	2019	55 days	Undirected	ESC	1	
Kitahara et al.	2020	6 or 10 weeks, cut in small pieces	SMAD and Wnt inhibition	ESC	1	> 85%
Wang et al.	2020	55 or 85 days	Undirected	ESC	1	
Shi et al.	2020	60 days	SMAD inhibition, cultured with HUVEC	ESC /iPSC	4	
Dong et al.	2020	40 days, sheared at 20 and 30d	Undirected	ESC /iPSC	2	
Bao et al.	2021	58 days	Undirected	ESC	1	
Revah et al.	2022	30–60 days	SMAD inhibition	iPSC	10	81%
Wilson et al.	2022	7–9 weeks	SMAD inhibition	iPSC	1	
Huang et al.	2022	7 days	Undirected	iPSC	1	
Kim et al.	2022	8 weeks	Undirected	ESC	1	
Jgamadze et al.	2023	80–88 days	SMAD inhibition	ESC/iPSC	3	82.1%
Zheng et al.	2023	10/15/25 days, cut in pieces	Midbrain	iPSC	3	Low for 25d org
Cao et al.	2023	50 days	Undirected	ESC/iPSC	2	100%
Schafer et al.	2023	52 days	SMAD inhibition + hiPSC-derived EMP	ESC/iPSC	7	
Cao et al.	2023	50 days	MGE	iPSC	1	
Chen et al.	2024	---	SMAD and Wnt inhibition	iPSC	6	

EC: endothelial cells. HUVEC: human umbilical vein endothelial cells. EMP: erythromyeloid progenitors. MGE: medial ganglionic eminence. ESC: embryonic stem cells. iPSC: induced pluripotent stem cells

Another factor to consider is the differentiation protocol employed to generate the COs. More than half of the published studies utilized an undirected approach, while more recent studies have used directed COs. In their experiments Zheng et al. [132] injected directed midbrain COs into the mouse striatum to ameliorate the phenotype of PD. In this case, post-mitotic markers of dopaminergic neurons, such as tyrosine hydroxylase (TH), started to be expressed at 15 DIV. The researchers attempted transplantation of COs at 10, 15, or 25 DIV and selected 15 DIV as the ideal age for the surgery. In fact, COs transplanted at 10 DIV developed only few TH + cells, while most of the 25 DIV COs did not survive after the transplant. This last result is vastly different from what other researchers obtained with other types of COs, especially considering that most studies successfully engrafted organoids at 40 DIV and older. Further research testing different types of directed organoids will be necessary to determine if early transplantation is needed only for midbrain organoids or also in other cases. The area of the brain in which the organoid is transplanted can also influence and instruct its differentiation. Huang et al. [42] used 7 DIV cell-aggregates and injected them into the mouse striatum. Two mpt, these organoids had low expression of cortical markers but expressed striatal markers. Cao et al [14]. also noted that when undirected COs were transplanted in the junction between the infarct core and the peri-infarct zone of the cortex, CO cells appeared to achieve different fates depending on the injury microenvironment: in the peri-injury area, the graft was mainly composed of mature and immature glutamatergic neurons, while in the infarct core the majority of the cells expressed the glial marker GFAP. This influence of the local environment on cell differentiation might also be relevant for some models of focal TBI, where there are marked differences between the lesioned tissue and the peri-lesion area. When the same group repeated the transplantation experiment using medial ganglionic eminence organoids, a consistent percentage of cells expressed markers of GABAergic interneurons [13].

Finally, the outcome of CO transplantation could be influenced by the specific cell line used in experiments. Despite reports demonstrating that organoids differentiate in a highly reproducible way from different lines [118], inter-line differences may be more pronounced in the in vivo context. For example, Jgamadze et al. [46] performed some of their transplantation experiments with 3 different cell lines (2 hiPSC and one hESC line). Results were similar under many parameters, such as graft survival and apoptosis, but one of the lines yielded significantly smaller grafts with less CD31^+^ structures. These results underline the importance to demonstrate protocol replicability by using more than one pluripotent cell line in experiments. Moreover, line-dependent differences could be particularly relevant for the use of organoid transplantation in personalized medicine.

In sum, grafting of human COs into animal models is a very active and developing area of research, and with the benefits of the more complex models the potential technical sources of variability also increase. So far, the labour- and cost-intensive protocols also have restraint sample sizes to expand in order to control for this variability. However, within the current technical challenges, as discussed below, it is therefore important to emphasize standardizing result reporting to enhance the reproducibility of the research.

### Current limitations

The research summarized here offers a hopeful preview of the potential of CO transplantation in regenerative medicine and modelling of the human pathologies, particularly focusing on TBI. Despite the promising results, some considerations must be taken into account regarding both applications, and the xenotransplantation protocols in general. We discuss these in the following sections.

### Cell replacement therapy

Concerning TBI, there is an indication that CO transplantation contributes to recovery through a dual mechanism, providing exogenous tissue repair and stimulating endogenous neurogenesis [121]. However, none of the current studies on TBI tried to discern the contribution of each of these processes to the functional recovery of the animals. Thus, it is not possible to establish if the CO cells played a direct role in the reconstruction of the host neural network. The results from Cao et al [14] (stroke model) hint indeed to the organoid neurons contributing to motor function restoration, but this should be verified in future studies more pertinent to TBI.

Another critical consideration is the potential clinical translation of the transplantation approach for treating injuries. The invasive surgery needed for organoid implantation in the brain would be justifiable primarily in cases of severe injuries or those already requiring surgery. Given that over 70% of reported brain injuries are mild TBI [15], with symptoms often resolving spontaneously, the necessity of such invasive procedures may be limited for most TBI cases. Additionally, the time required to generate patient-derived iPSCs and cultivate COs would preclude transplantation during the acute phases of trauma. Transplantation after the formation of a glial scar might pose challenges to effective integration, as demonstrated in prior experiments using foetal tissue [104]. The availability of quality-controlled iPSCs lines in biobanks [129] or the establishment of hypoimmunogenic iPSCs, which would allow allogenic transplant without need for immunosuppression [24, 25, 37, 41], could greatly reduce time requirements and costs. However, the protocol to grow organoids from stem cells would still need to be performed immediately before transplantation. An intriguing possibility is the establishment of organoid biobanks for general use, similar to organ transplantation protocols applicable during the acute phase following severe TBI.

With the current technical challenges, it seems that organoid transplantation would have a greater potential in the treatment of chronic neurodegenerative diseases for which there are few to nontherapeutic options available, such as PD. Cell replacement therapy has been long considered an attractive possibility in PD, and transplantation of stem cells-derived dopaminergic progenitor cells has reached clinical stage in recent years [8, 54, 110]. A first report from a single patient transplanted with autologous cells showed potential benefits of the treatment [96]. While protocols for deriving midbrain organoids from iPSCs are already established [47, 74], extensive pre-clinical testing will be required to ascertain that their transplantation could achieve similar benefits to that of dopaminergic precursors cells, and that they are comparably safe. For example, a major concern for safe clinical application would be the oncogenic potential of COs. Proliferative, undifferentiated cells were abundantly present in the organoids at time of transplantation and often continued to be detected months after transplantation [14, 46, 55]. In the study from Kitahara et al. [55] 6-weeks-old COs caused graft overgrowth when transplanted in both young and adult mice and they still contained more proliferative cells than 10-weeks organoids at 12 wpt. Excessive growth could cause increase of intracranial pressure and have a negative effect on the host brain. Therefore, it seems that the choice of the appropriate in vitro age of the organoids for transplant would be a trade-off between efficiency of survival and integration and safety in a clinical setting. Further studies are required to better investigate the potential for malignant growth of the grafted COs.

### Disease modelling

We have here presented examples of how in vivo COs can be used to model human pathologies, a technique that offers great potential also for TBI studies. However, also for this application some caveats have to be taken in account.

In vitro COs partially recapitulate the organization of the cortex in cellular layers [59] and when transplanted they still express markers of different neuronal layers. However, several groups reported the loss of laminar structure after transplantation [20, 46, 86]. Given this observation, it seems unlikely that transplanted organoid could develop in properly structured human brain tissue, including gyrification. Previous studies of human brain organoid models offer varying results regarding presence and degree of gyrification. As reviewed by Scott et al. [97], different strategies are being explored in order to improve the folding in brain organoids. A gyrification resembling the human brain is desirable in translational studies: injury patterns in human TBI are often anatomically associated with sulci and gyri. For instance, early neuropathological signs of chronic traumatic encephalopathy (CTE), perivascular tau depositions, appear at deep locations in cortical sulci [1]. However, the mechanism for this phenomenon is largely unknown, and it could be the result of microvessel anatomy rather than of the convolution of the cortical surface. Accordingly, lissenchaphalic species, such as rodents, recapitulate some of the hallmarks of CTE and other TBI-associated pathology observed in humans [11, 48]. In addition, CTE-associated pathology is also observed in deeper parts of the brain [11] and can therefore, in theory, be studied in organoids to some extent.

Despite showing increased maturation in cell morphology and transcriptomics compared to in vitro COs, the transplanted COs still resembled late foetal or at most early post-natal human tissue, even after long periods in vivo [86, 93]. This is in line with previous studies that demonstrated that COs in vitro follow the timeline of human foetal development, acquiring a postnatal transcriptomic signature between 250 and 300 days in culture [36]. The limited life span of the species commonly used for xenotransplantation implies that it would be difficult to reach later stages of human tissue development. Consequently, this model would most likely not be suitable for modelling the ageing human brain. This is particularly relevant for TBI, since older people have a higher incidence of head trauma [7]. It would also be difficult to try and study the association between TBI and neurodegenerative diseases [12], since that would require faster tissue development or long-term longitudinal studies. However, known genetic predispositions to developing CTE or neurodegenerative diseases following TBI can be studied in this model.

Another factor to consider when modelling human disease is that the long-term transplantation of human cells in other species requires suppression of the host immune system to avoid graft rejection. As such, it is not possible to replicate all aspects of the immune response to injury in this model. This is particularly problematic for modelling TBI, in which breakage of the blood brain barrier and infiltration of peripheral immune cells in the brain parenchyma play a significant role. A possible solution would be the use of hypoimmunogenic iPSCs, which could be transplanted into fully immunocompetent animals [25, 41]. On the other hand, this approach would require extensive genetic engineering of any cell line used to produce the organoids and would thus be labour-intensive.

### General concerns

Future research on TBI using chimera models should aim to provide more detailed and thorough descriptions and discussions of experimental parameters. For example, the transplantation protocol should not create additional tissue damage to the host brain after the initial injury, or it would be difficult to argue the validity of the model in replicating TBI. Accurate reporting of control group characteristics is crucial, including specifying the sex of animals involved. Ideally, experiments should be conducted in both male and female subjects. In publications covering CO transplantation in rodents, only a limited number included both male and female animals, and none considered sex as an experimental variable (see Table 1). Although sex may not directly impact the transplantation process, it is recognized that sexual differences exist in the response to TBI, as observed in both rodent models and clinical studies [39]. Therefore, considering sex as a factor is essential for a comprehensive understanding of transplantation outcomes. Given the early stage of this field, we emphasize the importance of standardizing result reporting in forthcoming papers to enhance the reproducibility of research. Building on experimental evidence from the literature on TBI, we recommend that certain parameters and relevant variables should be consistently and accurately reported in future research. These variables are suggested in Table 3.

**Table 3:** Relevant experimental variables

Animals	Organoids	Procedure
Sex	Number of cell lines used	Brain area used for transplant
Age	Detailed culture protocol	Surgical protocol (injury/no injury)
Immunosuppression	Age at transplant	Injury model, including precise description of sham controls
		Time between injury and transplant
		Time between transplant and outcome measure

Reporting of these parameters should be accurate and consistent to guarantee standardization and reproducibility of future studies

Finally, there are some ethical concerns regarding the implantation of human nervous tissue in other animals for research, which have been covered in several publications [9, 16, 23, 50, 60]. One point of discussion is the possibility that the human organoid could enhance the cerebral functions of the host animal, thus elevating its moral status and increasing the requirements for the protection of its welfare and dignity. We have seen how most of the presented experiments included the removal of host brain tissue or the creation of other types of injury before transplantation. In these cases, there was a loss of functionality whose recovery was aided by the presence of the graft, with ultimately no evidence of a benefit for the transplanted animals as compared to untreated controls. This was also confirmed by Mansour et al., who showed that transplanted mice were able to learn normally but performed worse than untreated controls when their spatial memory was tested [68]. Dong et al. [26] developed a protocol for the production of small COs which were then injected into the mouse medial prefrontal cortex with a micropipette, without further injury to the host tissue. Grafted animals exhibited normal behaviour in the open field test, but they showed an increase in percentage of freezing in a fear conditioning set-up. This result is contradicted by Revah et al. [86] who also injected their COs with a needle into the rat S1 cortex and observed no significant difference in performance in the open field, novel object recognition and fear conditioning tests. The difference in findings might be due to the brain area in which the organoids were implanted. In summary, there is currently limited evidence of potential enhancements in cerebral function in host animals. However, caution is advised when selecting the transplant site, especially when working with species to which a higher moral status than rodents is attributed. Among the studies presented, only one [55] involved transplantation in non-human primates, specifically in the motor cortex to avoid impacting higher brain functions. However, no behavioural observations were conducted.

As the field of brain organoid transplantation is expected to advance and overcome technical limitations, ethical thresholds should be established in anticipation of future scientific advances.

## Conclusions

In summary, the last six years have witnessed significant advancements in the development of a novel scientific model, allowing for the study of human nervous tissue in an in vivo system. Human COs have demonstrated the capability to integrate into the host animal brain and contribute to neural activity to a certain extent. Although primarily explored for regenerative medicine with promising outcomes, CO transplantation exhibits substantial potential for modelling human pathologies. We here highlighted certain characteristics that could offer advantages over traditional animal models or CO in vitro models, despite inherent limitations. Considering the challenges in translating pre-clinical findings from animal studies to clinical applications, we propose that the application of CO transplantation in TBI research holds promises and could provide valuable new insights.

By integrating human-specific organoids into a rodent model, one can closely mimic the complex interactions between human genetic factors and TBI outcomes. This innovative approach allows for a more comprehensive exploration of the impact of human genetics on injury response and gene function in TBI pathology within an in vivo system. The human organoid in rodent chimera model provides a unique opportunity to bridge the translational gap between preclinical research and potential therapeutic applications for human brain injuries. Moreover, beyond its applications in regenerative medicine and modeling human pathologies, CO transplantation also holds potential for drug testing. The integration of COs into the host brain provides a unique platform to study the effects of pharmaceutical compounds on human neural tissue in a more physiologically relevant context. This additional dimension further enhances the versatility and utility of CO transplantation as a valuable tool in scientific research.

## Data Availability

Not applicable.
